# Intransparent German number words complicate transcoding – a translingual comparison with Japanese

**DOI:** 10.3389/fpsyg.2015.00740

**Published:** 2015-06-11

**Authors:** Korbinian Moeller, Julia Zuber, Naoko Olsen, Hans-Christoph Nuerk, Klaus Willmes

**Affiliations:** ^1^Knowledge Media Research CenterTuebingen, Germany; ^2^Department of Psychology, University of TuebingenTuebingen, Germany; ^3^LEAD Graduate School, University of TuebingenTuebingen, Germany; ^4^Section Neuropsychology, Department of Neurology, RWTH Aachen UniversityTuebingen, Germany

**Keywords:** transcoding, German, Japanese, number-word system

## Abstract

Superior early numerical competencies of children in several Asian countries have (amongst others) been attributed to the higher transparency of their number word systems. Here, we directly investigated this claim by evaluating whether Japanese children’s transcoding performance when writing numbers to dictation (e.g., “twenty five” → 25) was less error prone than that of German-speaking children – both in general as well as when considering language-specific attributes of the German number word system such as the inversion property, in particular. In line with this hypothesis we observed that German-speaking children committed more transcoding errors in general than their Japanese peers. Moreover, their error pattern reflected the specific inversion intransparency of the German number-word system. Inversion errors in transcoding represented the most prominent error category in German-speaking children, but were almost absent in Japanese-speaking children. We conclude that the less transparent German number-word system complicates the acquisition of the correspondence between symbolic Arabic numbers and their respective verbal number words.

## Introduction

Recent years have witnessed increasing research interest in the impact of specific language properties on numerical development. A large proportion of these studies focused on the comparison of Western (mostly European and American English) and Asian (mostly Korean, Japanese, and Chinese) children’s performance in mathematics. Contrasting these different languages and their cultural backgrounds revealed impressive differences in favor of children from those Asian countries (e.g., Stevenson et al., 1985; Stigler et al., 1987; Miura et al., 1999). For example, Geary et al. (1992) found that Chinese first graders were faster and more accurate in addition tasks than matched US children. Similarly, superiority in subtraction performance of Korean children over US children was reported (Song and Ginsburg, 1987; Fuson and Kwon, 1992). However, these differences are not restricted to more complex mathematical tasks like mental calculation. Even in basic numerical tasks such as counting or place-value understanding differences favoring Asian children were observed (mostly Chinese children: Miura et al., 1988; Miller et al., 1995). Several reasons have been proposed to explain this superiority of children in several Asian countries. On the one hand, various cultural differences have been mentioned, including variations in home experiences (e.g., greater parental expectations; Song and Ginsburg, 1987; Stevenson and Lee, 1990) as well as differences of educational systems (e.g., quality and quantity of mathematics instruction, rigor, or structure of the mathematics curriculum; Stevenson et al., 1985, 1987; Stigler et al., 1987; Chen and Stevenson, 1989; Hess and Azuma, 1991; Perry et al., 1993; Stevenson and Nerison-Low, 2000). However, it has to be considered that superior performance in basic numerical tasks was already reported before schooling or formal education starts (e.g., Stevenson et al., 1987), questioning the influence of schooling as the only relevant factor (see Miller et al., 2005 for a review).

As a consequence, it was also suggested that specific language characteristics such as the higher transparency of the number word systems of east-Asian languages, such as Japanese and Chinese, and their consistent reflection of the place-value structure of the Arabic number system might also have an impact on mathematics performance (Miura et al., 1988; Geary et al., 1992; Miller et al., 1995; Miura and Okamoto, 2003; see Ng and Rao, 2010 for a recent review; but see Ackerman, 1988 for limitations of this view).

In our view, two approaches may help to differentiate influences of language from those of culture more generally. First, language influences may be examined within the same culture and educational system. For instance Imbo et al. (2014) compared transcoding performance in Dutch- and French-speaking children in Belgium and observed advantages for French-speaking children (see also Dowker and Lloyd, 2005; Dowker et al., 2008; Colomé et al., 2010; Pixner et al., 2011; Salillas and Carreiras, 2014 for studies following this approach). Second, one might aim at considering the specificities of certain languages. Rather than just showing that Japanese or Chinese children are somehow and/or generally better in basic numerical and/or arithmetic tasks than their Western (e.g., German or English) peers, it would be instructive to show that they perform specifically better on those stimuli within the same task, for which the transparency of their number word system gives them a particular advantage. Vice versa, for stimuli for which the Japanese or Chinese number word system provides no particular advantage, differences should be smaller or non-existent at all. Importantly, general cultural differences cannot easily explain such differential effects, when differences between groups can be observed exclusively or predominantly for stimuli which differ with respect to specific attributes of the respective language systems.

In the current study we pursued this second rationale by investigating differences between Japanese and German children attending first grade of primary school regarding basic numerical abilities of transcoding and thus place-value processing. In the following, we will first briefly describe recent evidence concerning language influences on number processing before elaborating on the specific differences between the Japanese and German number word systems from which we derive our hypotheses.

### Language Influence on Numerical Performance

In general, the idea of a language-specific influence on numerical cognition is not new. Quite a few studies found that language-specific features influence performance in numerical tasks. For instance, Colomé et al. (2010) investigated influences of differences in number word formation between Spanish and Basque on adults’ addition performance. While Spanish number words reflect the base-10 structure of the Arabic number system, some Basque number words reflect a vigesimal base-20 structure. This means that number words are formed by combining multiples of 20 and units or teens (e.g., “36” is spoken as “hogeita hamasei” literally meaning “twenty and sixteen”). The authors observed that only Basque participants solved additions faster when they were presented as a multiple of 20 and a teen (e.g., 20 + 16) as compared to problems with the same results but emphasizing a base-10 structure composition [e.g., 26 + 10, see also Salillas and Carreiras (2014) for influences of Basque number words on number processing]. Moreover, language-specific influences on numerical performance have also been reported for children. For example, Seron and Fayol (1994) observed language influences comparing French- and Belgian-French-speaking children. In Belgium, decade structures like 70 and 90 are composed regularly [“septante” (“seventy”) and “nonante” (“ninety”)], whereas in French they are irregular [“soixante-dix” (“sixty-ten”) and “quatre-vingt-dix” (“fourty-twenty-ten”)]. When children were asked to write down numbers to dictation (e.g., transcoding verbal number words to the corresponding Arabic number), Belgian children committed fewer errors on the respective decades than French children. Moreover, for French-speaking children error types clearly reflected the verbal lexical primitives used to express these decades. For instance, “quatre-vingt-dix-sept” (“four-twenty-ten-seven,” which is the corresponding French number word for 97 = 4 ^∗^ 20 + 17) was written as 4217, 42017, or 8017 (see also Krinzinger et al., 2011, for a comparison of French, Dutch, and German; Göbel et al., 2014 for language influences on arithmetic).

Moreover, in several number-word systems (e.g., German, Dutch, Arabic, Maltese, Malagasy, etc., Comrie, 2005) tens and units are uttered in reversed order with respect to their order in Arabic notation (e.g., in German “21” is spoken as “einundzwanzig,” i.e., “one-and-twenty” translated literally) – referred to as the inversion property of number words. Interestingly, transcoding performance of German-speaking children was found to be severely influenced by the inversion property of German number words. In fact, about 50% of transcoding errors of German-speaking first-graders were related to inversion (Zuber et al., 2009). In contrast, transcoding studies in languages without inversion (except for teen numbers, e.g., “thirteen” in English) did not specifically report inversion errors (e.g., French: Barrouillet et al., 2004; Camos, 2008; Italian: Power and Dal Martello, 1990, 1997). Different studies replicated this observation (e.g., Imbo et al., 2014, for a comparison of Dutch and French in Belgian children, see also Pixner et al., 2011 for a comparison of inverted and non-inverted number words in Czech). These findings provide first evidence that transcoding performance may somehow be related to language-specific features. However, those studies were restricted to a comparison among different European cultures.

While there are, to the best of our knowledge, no translingual studies directly contrasting transcoding in some Western and Asian number-word systems, there are some studies investigating the understanding of the base-10 place-value structure of the Arabic number system. In a first approach, Miura et al. (1988, 1994; Miura and Okamoto, 1989, 2003) assessed whether Asian (including Chinese, Japanese, and Korean) differed from Western (including French, Swedish, and US) children with regard to their representation of the base-10 place-value structure of the Arabic number system. They asked children to construct various numbers by using base-10-blocks. Indeed, children considered how their specific languages reflect or translate the place-value structure of the Arabic number system into their number words. Miura et al. (1988, 1994) suggested that better performance with regard to base-10 understanding of these Asian children is due to a strong influence of language, namely the more transparent correspondence of number words to the place-value structure of Arabic numbers in the respective languages. However, these findings were questioned in subsequent studies. Towse and Saxton (1997) demonstrated that English-speaking children showed similar base-10 place-value understanding as compared to the Asian samples investigated by Miura et al. (1988, 1994; including Chinese, Japanese, and Korean children) when instructed appropriately.

The current study picks up this argument and evaluates the account of Miura et al. (1988) explicitly. If Japanese children have better place-value understanding of the Arabic number system due to higher transparency of their number word system, they should commit less place-value related errors when transcoding number words into Arabic numbers. In particular, errors related to specific intransparencies in comparison to another number word system without these attributes should be examined. Therefore, the current study is designed to compare Japanese- and German-speaking children’s performance in a basic numerical transcoding task. Contrasting children’s performance in these two disparate number word systems should provide further insight into the extent to which language influences the acquisition of fundamental numerical abilities.

Before introducing our hypotheses in more detail, the structure of the Japanese and German number word system will be sketched briefly, to outline their peculiarities and their possible impact on number processing.

### Differences between the Japanese and the German Number Word System

Number word systems all over the world can differ in several aspects (e.g., base, order, etc.; Comrie, 2005). In several Asian languages, such as Japanese, the number word systems are very transparent. Japanese children only have to memorize the number names from one to nine and the multipliers “juu” (“ten”), “hyaku” (“hundred”), and “sen” (“thousand”), etc.; larger numbers are then generated according to a set of rules. Decade names are formed by multiplicative composition, e.g., 40 is “yon-juu” (“four–ten”), larger numbers combine multiplicative and additive composition, e.g., 48 is “yon-juu-hachi” (“four-ten-eight”). So there is a consistent relationship between number words and corresponding digits as well as the multiplier in the place-value structure of the Arabic number system for all multi-digit numbers. In Japanese, the order in which units, tens, hundreds, etc. are named in number words thus follows the corresponding order of Arabic digits in a multi-digit number. However, this is different in some Western languages such as German. Here, the order in which tens and units are uttered is inverted in teens and all other two-digit number words: e.g., 21 is pronounced as “one-and-twenty” (“twenty-one”).

Furthermore, in Japanese Arabic digits are named identically in number words irrespective of their position within the number (e.g., 2 → “two”; 20 → “two-ten”). In contrast, Arabic digits correspond to different number words at the tens position as compared to the unit position in German number words (e.g., 2 as the number word “two” vs. 2 in “twenty”). Finally, a third difference refers to the name of the multiplier. In Japanese, the multiplier is explicitly part of the spoken number word. For instance, 40 (4 ^∗^ 10) is spoken “yon-juu” (“four-ten”), and 400 (4 ^∗^ 100) is spoken “yon-hyaku” (“four-hundred”). In German, the multiplier is only transparent from three-digit numbers upward [e.g., 400 → “vier hundert” (“four-hundred”)], but intransparent for two-digit numbers [e.g., 40 → “vierzig” (“fourty”) instead of “vier-zehn” (“four-ten”) as in Japanese, see Ng and Rao, 2010 for a review on the influence of Asian number word systems].

However, there are also some intransparencies common to both languages. These concern the role of the digits 0 and 1 in three-digit number words. Both languages do not name “zero” at the tens place (e.g., “207” is “two-hundred and seven” and not “two-hundred-zero-ten-seven”). This intransparency might cause additive composition errors where either zero is left out (“two-hundred and seven” → 27) or the overwriting rule of zeros is ignored (“two-hundred and seven” → 2007). Similarly, “one” is not named at the tens position in both languages (“217” is named as “two-hundred-ten-seven” in Japanese and not “two-hundred-one-ten-seven”). Thus, there is only a multiplier (“ten”) for the tens digit, but no value for the digit itself. Therefore, the value of the corresponding Arabic digit cannot be determined from the number-word (e.g., no digit value named in a three-digit number word might as well reflect the value “zero” or “one”).

Taken together, these two number word systems differ in several aspects with the Japanese number word system being the more transparent one. If children’s errors are related to the specificities of their number word system when they translate one number format to another, this would be an indication that language influences numerical performance. Because the German number word system is rather intransparent compared to the Japanese one due to its inversion property, it is expected that German speaking children commit more errors reflecting their problems with understanding the place-value structure of Arabic numbers. Generally, this refers to errors violating the syntactic structure of the respective multi-digit number (see Materials and Methods section for a taxonomy of transcoding errors) such as additive and multiplicative composition errors (e.g., “two-hundred seven” → 2007) as well as inversion errors. As described above there are commonalities and differences between German and Japanese with respect to transparency in additive and multiplicative composition. Nevertheless, because digits correspond to specific number names at the tens position (e.g., 2 → “twenty”) and the fact that the multiplier is not indicated in German number words denoting the decades, we expected more additive and multiplicative transcoding errors for German- as compared to Japanese-speaking children. Importantly, however, the inversion property and associated inversion errors are of highest interest in this study because there is no number word inversion in Japanese at all. In Contrast, German children’s transcoding errors have been found to be inversion related in 50% of the cases [i.e., “twenty-five” (spoken as “five and twenty”) → 52, Zuber et al., 2009], thereby reflecting a number-word specific intransparency. As no inversion of tens and units is present in the Japanese number word system, no such errors should occur in Japanese-speaking children. Thus, given an influence of language on performance, error rates should not only differ in general, but should also be differentially related to specific attributes of the number word structure of the respective languages.

## Materials and Methods

### Participants

In total, 40 children participated in the study. Twenty German-speaking children (10 girls), were recruited from a German elementary school, mean age was 7.32 years (SD = 0.36; range 6 years 7 months to 7 years 8 months). All children spoke German as their native language, none of them had been noted for having specific difficulties in mathematics or other school problems. Additionally, twenty Japanese children (seven girls) were recruited from a Japanese elementary school in Germany. Their mean age was 7.27 years (SD = 0.36; range 6 years 5 months to 7 years 7 months). Japanese schools in Germany follow the Japanese curricula and teaching is exclusively in Japanese. Moreover, all children’s parents were both native speakers of Japanese, and only Japanese was spoken at home. Additionally, Japanese children did not speak any German nor had they encountered German numbers, yet. According to the respective school curricula the number of mathematics classes is equal for both language groups. By the end of first grade, all children should know the numbers up to 20 and be able to perform simple additions and subtractions within this range. To furthermore ensure an equal level of education, both groups were tested toward the end of the academic year, this means German-speaking children at the end of May and Japanese-speaking children in February, because the Japanese academic year ends in March.

The study was approved by the local school authorities and carried out in line with the latest version of the Declaration of Helsinki. Written informed consent was obtained from parents of all participating children prior to the study.

### Tasks and Stimuli

The *transcoding* task consisted of 67 stimuli (i.e., 9 single-digit, 36 two-digit, and 22 three-digit numbers), incorporating all lexical primitives and different syntactic structures. Children had to write them down as Arabic numbers to dictation. Numerical structures not yet learned at school (i.e., three-digit numbers) were presented in order to assess whether children were able to apply and generalize rules they had already learned on simpler forms (see Byrge et al., 2014 for kindergartner’s writing down three-digit numbers). The order of the stimuli was randomly assigned, but the task always started with a one-digit number.

There was also a block of items, in which children had to read aloud Arabic numbers. However, as the results did not differ substantially between these two conditions and the error analysis of the reading aloud condition is less discriminating (e.g., no child would name 324 as “thirty thousand twenty four” but when instructed to write down “three hundred twenty four” in Arabic notation 30024 is a quite common error) this article focuses on the results of the writing to dictation condition.

### Procedure

Children were tested individually in a quiet room during school hours in one-on-one sessions. Children had to write down numbers to dictation on a blank sheet, one below another. No feedback was given as to the correctness of the results. The critical 67 trials were preceded by two practice trials to familiarize children with the task.

### Transcoding Error Analysis

Errors were categorized according to the taxonomy used in Zuber et al. (2009; extended and slightly modified from Deloche and Seron, 1982). This categorization is used because it allows classification of inversion errors; moreover, it is kept as general as possible to enable its use in a variety of languages.

In general, this categorization distinguishes *lexical* from *syntactic* errors (following Deloche and Seron, 1982, 1987). Lexical errors concerned the substitution of one (or more) lexical elements by another one with no modification of the syntactic structure. This error category was subdivided into lexical value errors being (a) zero dependent, e.g., “eighty” → 81; or (b) zero independent, e.g., “thirty-four” → 35), and (c) lexical class errors, where the primitive itself is correct but its class is not (e.g., “eighty” → 18). Lexical errors that could not be classified into one of these categories were coded as (d) other lexical errors.

Errors were classified to be *syntactic* when they altered the syntactic structure of the produced numeral compared to the target form. This could either be due to violations of the (a) additive composition rule e.g., “three hundred twenty” → 30020 (when twenty is appended in the composition rather than added) and (b) multiplicative composition rule, e.g., “three hundred” → 3100 (when 100 is appended in the composition rather than multiplied. Further, (c) inversion errors were also categorized as syntactic errors as they mirror the understanding of syntactic rules (i.e., place-value structuring). Inversion errors could either be due to disregard of inversion meaning that the to-be-inverted digits were produced in the wrong order [e.g., “twenty-five” (“five and twenty”) → 52], or reflecting wrong application of inversion, this means, when hearing “three hundred,” children may wrongly apply the inversion rule (e.g., “three hundred” → 103) reflecting an overgeneralization of this rule. Again, errors that could not be classified into these subcategories were coded as (d) other syntactic errors.

Eventually, errors including both wrong lexical elements and incorrect syntactic structures were coded as *combination errors*.

Finally, transcoding errors that could not be classified as belonging to one of the categories specified above were coded as *other errors*.

## Results

Inferential statistics were conducted on arcsine-transformed error proportions to approximate normal distributions. In case the sphericity assumption was violated, the original degrees of freedom together with the respective Greenhouse–Geisser coefficients (GGs) are reported. One German-speaking child was excluded from further analyses because 24 of the 25 transcoding errors of this child were non-responses. For the remaining participants there were 2.2% non-responses in German speaking children and 0.3% in Japanese children, which were not included in the analyses.

### Overall Error Categories

To examine whether absolute error rates differed between the languages a 2 (language) × 4 (error categories: lexical, syntactic, combination errors, others) ANOVA was conducted. The ANOVA revealed significant main effects for both factors [language: *F*(1,37) = 31.72, *p* < 0.001, $\eta_{p}^{2}$ = 0.46; error category: *F*(3,111) = 38.66, *p* < 0.001, $\eta_{p}^{2}$ = 0.51, GG = 0.86] and a significant interaction [*F*(3,111) = 13.31, *p* < 0.001, $\eta_{p}^{2}$ = 0.27, GG = 0.86]. German-speaking children committed reliably more errors in general than their Japanese-speaking counterparts (7.2 vs. 1.4%, respectively). Additionally, the frequency of error categories differed significantly: pairwise contrasts indicated that syntactic errors were reliably more frequent than all other error categories (10.0%; all *p* < 0.001, Bonferroni-corrected) whereas there were no reliable differences between the remaining error categories (lexical errors: 1.7%, combination errors: 2.9%, other errors: 2.1%; all *p* > 0.9). The reliable two-way interaction indicated that languages differed reliably for the respective profile of error categories (see **Table 1A**). To evaluate our hypothesis that differences should be most pronounced for syntactic errors, we conducted three additional two-way ANOVAs with the factors language group and error category in which the latter reflected all possible pairwise combinations of the syntactic and one of the other error categories (i.e., syntactic vs. lexical errors; syntactic vs. combination errors, syntactic vs. other errors). To account for influences of multiple testing we reduced the alpha level accordingly (significant when *p* < 0.05/3 = 0.017). The ANOVAs consistently revealed reliable interactions of language group and error categories for syntactic vs. lexical errors [*F*(1,37) = 29.65, *p* < 0.001, $\eta_{p}^{2}$ = 0.45], syntactic vs. other errors [*F*(1,37) = 22.87, *p* < 0.001, $\eta_{p}^{2}$ = 0.38] as well as the interaction of language group and syntactic vs. combination errors [*F*(1,37) = 5.78, *p* = 0.021, $\eta_{p}^{2}$ = 0.14]. Importantly, these interactions indicated language differences for syntactic errors (14.0%; German: 17.4% vs. Japanese: 3.4%) to be more pronounced than those for lexical (1.8%; German: 2.7% vs. Japanese: 0.9%), other errors (2.0%; German: 2.3% vs. Japanese: 0.3%), and combination errors (6.0%; German: 6.1% vs. Japanese: 0.1%). Furthermore, simple effects indicated that language differences were reliable for all error categories with German-speaking children consistently committing more transcoding errors [syntactic: *t*(37) = 6.41, *p* < 0.001; combined [*t*(37) = 4.51, *p* < 0.001; lexical errors: *t*(37) = 2.36, *p* < 0.05; other errors: *t*(37) = 2.44, *p* < 0.05]. In sum, this corroborated our hypothesis that language differences should be most pronounced for syntactic errors, as these include inversion errors that should be specific to German-speaking children.

**Table 1:** Overview of absolute error rates for all error categories **(A)** as well as absolute and relative error rates for subcategories of syntactic **(B)** and lexical errors **(C)** separated for German- and Japanese-speaking children, SEM given in parentheses.

Error categories	German	Japanese
**(A) Absolute overall error rates**
Syntactic errors	17.4% (1.9)	3.4% (1.9)
Lexical errors	2.7% (0.7)	0.9% (0.7)
Combination errors	6.1% (1.5)	0.1% (1.4)
Other errors	2.3% (0.7)	0.3% (0.7)
**(B) Subcategories syntactic errors**
	**Absolute**	**Relative**	**Absolute**	**Relative**
Additive composition errors	7.2% (1.7)	33.3% (8.6)	2.9% (6.0)	75.8% (12.4)
Multiplicative composition errors	1.3% (2.0)	6.2% (2.0)	0.2% (1.0)	1.8% (2.8)
Inversion errors	8.6% (6.7)	58.5% (8.8)	0.3% (0.7)	22.2% (12.7)
**(C) Subcategories lexical errors**
Lexical value errors	0.5% (0.3)	9.7% (8.4)	0.1% (0.1)	20.0% (13.0)
Lexical value errors incl. zero	0.6% (0.5)	13.3% (10.9)	0.4% (0.3)	36.0% (16.9)
Lexical class errors	0.9% (0.4)	31.7% (10.5)	0.1% (0.1)	4.0% (16.2)

### Error Subcategories

To investigate whether these different error distributions were indeed specifically related to number word attributes the *absolute* and *relative* error frequencies of the subcategories of syntactic and lexical errors per child were evaluated in more detail.

We did not consider combination errors and other errors here because we had no specific hypothesis for language effects on the latter. Regarding combination errors, Japanese-speaking children did not commit any error in three of the four categories of combination errors we observed (i.e., combination of lexical and inversion errors, lexical, syntactic and inversion errors, as well as syntactic and inversion errors). For the remaining combination of lexical and syntactic errors there was only one Japanese child who committed one such error. Therefore, we refrained from further analyzing frequencies of error subcategories of combination errors.

To allow applicability of ANOVA methods for the patterns of *relative* error frequencies, we excluded one subcategory of errors each from the analyses to avoid complete dependency among error categories in that they would always sum up to 100%. We eliminated the categories ‘other syntactic errors’ and ‘other lexical errors’ because they were of only marginal theoretical interest. Thus, relative error frequencies do not add up to 100%. To keep the analyses of absolute and relative error rates comparable we also excluded the categories ‘other syntactic errors’ and ‘other lexical errors’ when analyzing the absolute rates of error subcategories. This means that the analysis for syntactic errors discerned the subcategories inversion errors, as well as additive and multiplicative composition errors. On the other hand, the analysis for lexical errors discerned the subcategories of lexical class errors, lexical value errors not including and lexical value errors including zero.

Moreover, for the analysis of relative frequencies all children who did not commit at least one error were excluded from analyses since they do not contribute to potential differentiation between error subcategories. For syntactic errors, this affected 11 of the Japanese-speaking children and no German-speaking child. For lexical errors this affected 15 Japanese- and 7 German-speaking children. Because of this considerable reduction of sample sizes and the generally low frequencies of lexical errors the results for the specific evaluation of lexical error subcategories need to be treated with caution.

#### Syntactic Errors

##### Absolute error rates

The 2 (language group) × 3 (error subcategory: additive composition, multiplicative composition, inversion) ANOVA revealed a reliable main effect of language group [*F*(1,37) = 37.34, *p* < 0.001, $\eta_{p}^{2}$ = 0.34] indicating that German-speaking children committed significantly more syntactic transcoding errors across all subcategories than their Japanese-peaking peers (5.7% vs. 1.1%, respectively). Additionally, the main effect of error subcategory was reliable [*F*(2,74) = 10.47, *p* < 0.01, GG = 0.69; $\eta_{p}^{2}$ = 0.22] suggesting that error rates were not distributed equally across syntactic error subcategories. Pairwise comparisons showed that multiplicative composition errors were reliably less frequent (0.8%, both *p* < 0.05, Bonferroni corrected) than both additive composition (5.1%) and inversion errors (4.4%). This error pattern was further qualified by language as indicated by the significant interaction between language group and error subcategory [*F*(2,74) = 7.50, *p* < 0.01, GG = 0.69, $\eta_{p}^{2}$ = 0.17, see **Table 1B**]. As we hypothesized that language differences within the category of syntactic errors should be driven by the specifically increased frequencies of inversion errors in German-speaking children, we conducted two additional two-way ANOVAs with the factors language group and error subcategory. The factor error subcategory reflected the pairwise combinations of inversion errors with other syntactic error subcategories (i.e., inversion vs. multiplicative composition errors; inversion vs. additive composition errors). We reduced the alpha level accordingly to control for influences of multiple comparisons (i.e., significant when *p* < 0.05/2 = 0.025). The ANOVAs revealed a marginally reliable interaction of language group and error subcategories inversion vs. additive composition errors [*F*(1,37) = 4.80, *p* < 0.05, $\eta_{p}^{2}$ = 0.12] whereas the interaction for error subcategories inversion vs. multiplicative composition errors was highly significant [*F*(1,37) = 30.44, *p* < 0.001, $\eta_{p}^{2}$ = 0.45]. Importantly, the former interaction indicated that the language difference for inversion errors (8.4%; German: 8.6% vs. Japanese: 0.2%) tended to be more pronounced than that for additive composition errors (4.3%; German: 7.2% vs. Japanese: 2.9%). Moreover, the language difference was significantly more pronounced for inversion errors than for multiplicative composition errors (1.1%, German: 1.3% vs. Japanese: 0.2%).

Tests for simple effects indicated that absolute frequencies of error subcategories were significantly higher for German-speaking children for all error subcategories [inversion errors: 8.6% vs. 0.2%, *t*(37) = 8.55, *p* < 0.001; additive composition errors: 7.2% vs. 3.0%, *t*(37) = 2.04, *p* < 0.05; multiplicative composition errors: 1.3% vs. 0.2%, *t*(37) = 2.62, *p* < 0.05].

##### Relative error rates

As to be expected, the 2 (language group) × 3 (error subcategory: additive composition, multiplicative composition, inversion) ANOVA on relative error frequencies revealed no significant main effect of language group [*F*(1,27) < 1]. However, the main effect of error subcategory was reliable [*F*(2,52) = 10.96, *p* < 0.001,; $\eta_{p}^{2}$ = 0.30, GG = 0.57] suggesting that syntactic error subcategories were not distributed equally. Pairwise comparisons showed that multiplicative composition errors were reliably less frequent (4.0%, both *p* < 0.05, Bonferroni corrected) than both additive composition (54.6%) and inversion errors (40.4%). Importantly, however, this error pattern was qualified by language as indicated by the reliable interaction between language group and error subcategory [*F*(2,52) = 7.59, *p* < 0.01, $\eta_{p}^{2}$ = 0.23, GG = 0.57, see **Table 1B**]. To evaluate whether language differences within the category of syntactic errors were indeed driven by the specifically increased frequencies of inversion errors in German-speaking children, two additional two-way ANOVAs with the factors language group and error subcategory were carried out. The latter factor reflected the pairwise combinations of inversion errors with other syntactic error subcategories (i.e., inversion vs. multiplicative composition errors; inversion vs. additive composition errors). To account for influences of multiple testing we reduced the alpha level accordingly (significant when *p* < 0.05/2 = 0.025). The ANOVAs revealed a reliable interaction of language group and error subcategories inversion vs. additive composition errors [*F*(1,26) = 8.00, *p* < 0.01, $\eta_{p}^{2}$ = 0.24] whereas the interaction with error subcategories inversion vs. multiplicative composition errors was not reliable [*F*(1,26) = 3.12, *p* = 0.09, $\eta_{p}^{2}$ = 0.11]. Importantly, the former interaction indicated that the language differences for inversion errors (36.3%; German: 58.5% vs. Japanese: 22.2%) were indeed more pronounced than for additive composition errors (-42.6%; German: 33.3% vs. Japanese: 75.8%).

Tests for simple effects substantiated that relative frequencies of the error subcategories were significantly higher for German-speaking children for inversion errors [*t*(26) = 2.56, *p* < 0.05], whereas Japanese-speaking children committed relatively more additive composition errors [*t*(26) = 3.02, *p* < 0.01]. There was no reliable difference for multiplicative composition errors [*t*(26) = 1.46, *p* = 0.16].

Taken together, the frequencies of absolute and relative syntactic error subcategories mirrored the hypothesized language specificities. The inversion property of German number words led to a specific absolute but also relative increase of inversion errors not present for Japanese-speaking children. However, these data also indicate that additive composition errors were relatively more prominent in Japanese-speaking children – even though they were more prominent in absolute terms for German-speaking children.

#### Lexical Errors

##### Absolute error rates

The 2 (language) × 3 (lexical errors: zero dependent, zero independent, lexical class) ANOVA revealed no reliable effects of the factors error subcategories [*F*(2,74) < 1] and language group [*F*(1,37) = 2.86, *p* = 0.10, $\eta_{p}^{2}$ = 0.07, GG = 0.89] nor a significant interaction of these two factors [*F*(2,74) = 1.06, *p* = 0.35, $\eta_{p}^{2}$ = 0.03, GG = 0.89, see **Table 1C**].

Simple effects revealed that German-speaking children committed significantly more lexical class errors than Japanese-speaking children [0.9 vs. 0.07%, *t*(37) = 2.04, *p* < 0.05]. In contrast, there were no reliable language differences for zero-independent [0.5 vs. 0.07%, respectively, *t*(37) = 1.30, *p* = 0.21] and zero-dependent errors [0.6% vs. 0.4%, *t*(37) = 0.19, *p* = 0.85].

##### Relative error rates

The 2 (language) × 3 (lexical errors: zero dependent, zero independent, lexical class) ANOVA neither revealed reliable effects of the factors error subcategories [*F*(2,30) < 1] and language group [*F*(1,15) < 1] nor an interaction of these two factors [*F*(2,30) = 1.48, *p* = 0.24, $\eta_{p}^{2}$ = 0.09, see **Table 1C**].

In summary, this pattern is in line with our specificity hypothesis that language differences should be most prominent for syntactic error categories reflecting differences of the number word systems compared.

## Discussion

The aim of this study was to investigate influences of language on numerical development by means of contrasting German- and Japanese-speaking children’s transcoding performance by the end of first grade. We were particularly interested in whether there were only general differences in the overall performance level or rather specific error patterns for the two language groups, reflecting the specific intransparencies of the respective number word systems. In particular, we expected inversion errors to be more prominent in German-speaking children.

In line with our expectations we observed strong indications of language influences on transcoding performance. First, German-speaking children, who have to learn the less transparent number word system, committed reliably more transcoding errors in general. However, and more importantly, more fine-grained analyses corroborated our more specific hypothesis that the distribution across error-types should not be arbitrary, but reflect the specificities of the respective number word systems. German-speaking children showed higher absolute rates of syntactic transcoding errors in general and each subcategory of syntactic errors (i.e., inversion, additive, and multiplicative composition) in particular. This reflects less precise overall understanding of the composition of multi-digit numbers out of their single-digit components in German-speaking children. Additionally, within the category of syntactic transcoding errors consideration of absolute and relative error rates indicated that inversion errors were not only the most prominent syntactic error subcategory in German-speaking children but also reliably more prominent than in Japanese-speaking children. The difficulty arising from the inversion property of German number words is further illustrated by the fact that inversion errors were not restricted to errors associated with the order of tens and units and thus the to-be-inverted digits (e.g., “twenty five” → 52). Instead, about 25% of inversion errors in German-speaking children reflected an overgeneralization of the inversion rule to hundreds (e.g., “nine hundred” → 109). No such error was committed by Japanese-speaking children. This clearly indicated the influence of the inversion property (i.e., the inverted order in which tens and units are named in number words) as a particular language attribute, which is present in German but not in Japanese, on children’s place-value understanding. In sum, these data clearly corroborate the hypothesis that language influences numerical abilities. This point and possible reasons for the higher specific and unspecific error rates in German will be discussed in the following.

Essentially, about half of the errors of German-speaking children were related to the inconsistency of inversion, whereas hardly any inversion errors were committed by Japanese-speaking children. Therefore, the interpretation of these results is straightforward. As there is no inversion in Japanese, almost none of these errors occurred; whereas, once inversion is present, the error distribution reflects this intransparency of the number word system. This is in line with the results of Pixner et al. (2011) who investigated transcoding in Czech-speaking children. In Czech both non-inverted and inverted number words for two-digit numbers are used commonly. Thus, Pixner et al. (2011) were able to directly evaluate the influence of inversion on transcoding performance within the same children. Similar to the present results, Pixner et al. (2011) observed that Czech children committed inversion related transcoding errors only when dictated number words were in the inverted format. However, transcoding errors of German-speaking children were not related exclusively to the specific attribute of inversion in German number words. Instead, error frequencies seemed to reflect the generally higher intransparency of the German number word system with regard to the reflection of the place-value structure of the Arabic number system: absolute frequencies of all subcategories of syntactic (place-value) errors were higher for German-speaking children.

In this respect, it is important to note that the few errors observed in Japanese children were often related to the only intransparency of the Japanese number-word system, such as the missing digit value for “one” (“one” is not named in the decade position, e.g., 217 → “two-hundred-ten-seven” and not “two-hundred-one-ten-seven”) or the missing digit and multiplier values for zero (e.g., “207” is “two-hundred and seven” and not “two-hundred-zero-ten-seven”). These intransparencies are related to additive composition. Accordingly, additive composition transcoding errors had a higher relative frequency in Japanese-speaking than in German-speaking children (even though German-speaking children committed more additive composition errors in absolute terms). Indeed, when examining the errors of Japanese children, it appears that almost all errors were related to the inconsistency of additive composition, whereas there were much fewer errors in all other error categories. Similarly, German speaking children’s additive composition errors constitute the major error subgroup besides inversion errors. Taken together, this supports the hypothesis of a language-specific influence of number word formation on transcoding performance.

However, when comparing error rates for additive composition rules between the two languages, one might wonder, why the absolute error rates of German-speaking children were about four times higher than those of Japanese-speaking children, even though they reflect the same additive composition principle. To account for these findings, the cognitive processes necessary for transcoding should be considered. Generally, the present pattern of results indicated that more transparent number word structures are less error prone, when children have to transcode numbers. But, additionally, another process might be involved as well. Because the structure of Japanese number words is simpler, it may require less working memory (WM) capacity to correctly transcode numbers. Indeed, WM was observed to be an influencing factor in several studies. For instance, Barrouillet et al. (2004) found WM to reliably predict transcoding performance (see also Camos, 2008). Moreover, Zuber et al. (2009) found that WM capacity was specifically important for transcoding in a language with inversion (see also Imbo et al., 2014). Therefore, one might speculate that an intransparent number word system requires more WM capacity and might therefore be more error prone in general. In contrast, a more transparent number word structure like the Japanese would require less WM capacity and may thus be less susceptible to WM capacity limitations. In this respect, WM capacity limitations in children may be partially responsible for our finding that German children committed more additive composition errors than Japanese children, even though the same principle has to be applied in the two languages.

Although this study revealed reliable influences of language on children’s numerical performance, one cannot exclude the possibility of other factors entirely. It should be acknowledged that even if teaching curricula were the same in both groups, school and home-related factors might have influenced children’s performance as well. In Japan mathematics performance is considered more important than in Western cultures (e.g., Stevenson and Lee, 1990) and children are trained and supported to a greater extent by parents and teachers (e.g., Song and Ginsburg, 1987; Stevenson and Lee, 1990). However, for first grade children, our data indicated that these factors do not seem to be the only ones to influence numerical performance because they can only account for better overall performance of Japanese children. Yet, we also observed specific differences in the distribution of absolute and relative error patterns of the two language groups, which corresponded very closely to the specificities of the two number word systems: German children did not only produced consistently more errors in absolute terms, but also showed higher absolute and relative rates of errors specifically related to the particular intransparencies of their number-word system regarding place-value coding (i.e., the inversion property). These specific effects cannot be explained by an account stressing general differences in learning, education or culture.

Finally, the impact of these language-specific influences on the acquisition of more complex numerical and arithmetical skills has to be considered. There is accumulating evidence that the understanding of basic numerical concepts including the place-value structure of the Arabic number system influences basic numerical (e.g., Holloway and Ansari, 2009; Moeller et al., 2009, 2015; Helmreich et al., 2011; Pixner et al., 2011) but also arithmetic performance (e.g., Levine et al., 1992; Kaufmann et al., 2003; Booth and Siegler, 2008; Göbel et al., 2014). On a very basic level, Cankaya et al. (2014) observed that the regular and transparent Turkish number word structure led to faster acquisition of counting principles and thus better counting performance in Turkish-speaking kindergartners (but see Vasilyeva et al., 2015 for a diverging account). For primary school children Moeller et al. (2011) found specific longitudinal influences of early place-value understanding (as assessed by transcoding performance amongst others) on children’s numerical development. The authors observed that children who committed more inversion-related transcoding errors at the end of grade 1 not only showed poorer addition performance at the end of grade 3 but also had particular difficulties solving addition problems requiring a carry and thus posing increased demands on their place-value understanding. This is further corroborated by data of Moura et al. (2013), who found that children with mathematical difficulties in middle grades of primary school had particular problems acquiring the syntactic transcoding rules allowing for correct place-value coding of multi-digit numbers. Additionally, Imbo et al. (2014) observed that Dutch second graders who experienced transcoding problems were also found to achieve generally worse in math as indicated by their grades.

Given this strong influence of language on the understanding of the place-value structure of the Arabic number system and the observed relation of the latter with mathematics performance more generally, the present results might also be informative with regard to the repeatedly observed performance difference in mathematical achievement between Western and Asian children (e.g., Fuson and Kwon, 1992). The present data suggest that it is more demanding for children to successfully acquire the relation between symbolic Arabic numbers and number words and thus to acquire place-value understanding in languages with intransparent number word systems. However, the studies described above indicated that such basic place-value understanding is predictive for successful acquisition of more complex arithmetic and mathematical competencies (e.g., Moeller et al., 2011 for the specific case of transcoding). Considering this state of affairs, the implications for teaching are straightforward: for children having to learn an intransparent and complex number word system it might be particularly important to teach and train the correspondence of the Arabic number system and number words more intensively, until children successfully master this link (e.g., Link et al., 2014).

## Conclusion

This study showed that German-speaking children were outperformed by Japanese-speaking children not only with respect to overall transcoding performance, but also experienced a particular disadvantage related to specific intransparencies of the German number word system. German-speaking children showed higher absolute error rates in general but also higher absolute and relative error rates specifically reflecting the inversion property of the German number word system. Such a differential performance pattern cannot be explained easily by general cultural accounts emphasizing the role of different learning cultures and/or education. Instead, these results are well in line with language accounts (Miller et al., 2005 for a review) suggesting that transcoding performance should be affected most where the respective number word system is most intransparent.

From this we conclude that the intransparency of the German number word system hampers fast and accurate acquisition of the correspondence between symbolic Arabic numbers and their verbal number names, while the transparency of the Japanese number word system leaves Japanese-speaking children at a considerable advantage. In sum, a better understanding of the difficulties imposed by the specificities of a particular number word system may help to corroborate transcoding skills and thus children’s place-value understanding, which – in turn – has been shown to predict future numerical and arithmetic achievement.

## Conflict of Interest Statement

The authors declare that the research was conducted in the absence of any commercial or financial relationships that could be construed as a potential conflict of interest.

## References

[B1] Ackerman P. L. (1988). Determinants of individual differences during skill acquisition: cognitive abilities and information processing. *J. Exp. Psychol. Gen.* 117 288–318. 10.1037/0096-3445.117.3.288

[B2] Barrouillet P. Camos V. Perruchet P. Seron X. (2004). ADAPT: a developmental, asemantic and procedural model for transcoding from verbal to Arabic numerals. *Psychol. Rev.* 111 368–394. 10.1037/0033-295X.111.2.368 15065914

[B3] Booth J. Siegler R. (2008). Numerical magnitude representations influence arithmetic learning. *Child Dev.* 79 1016–1031. 10.1111/j.1467-8624.2008.01173.x 18717904

[B4] Byrge L. Smith L. B. Mix K. S. (2014). Beginnings of place value: how preschoolers write three-digit numbers. *Child Dev.* 85 437–443. 10.1111/cdev.12162 24003873 PMC4445456

[B5] Camos V. (2008). Low working memory capacity impedes both efficiency and learning of number transcoding in children. *J. Exp. Child Psychol.* 99 37–57. 10.1016/j.jecp.2007.06.006 17854821

[B6] Cankaya O. LeFevre J. A. Dunbar K. (2014). The role of number naming systems and numeracy experiences in children’s rote counting: evidence from Turkish and Canadian children. *Learn. Indiv. Differ.* 32 238–245. 10.1016/j.lindif.2014.03.016

[B7] Chen C. S. Stevenson H. W. (1989). Homework: a cross-cultural examination. *Child Dev.* 60 551–561. 10.2307/1130721 2737007

[B9] Colomé A. Laka I. Sebastiaen-Galles N. (2010). Language effects in addition: how you say it counts. *Q. J. Exp. Psychol.* 63 965–983. 10.1080/17470210903134377 19742389

[B10] Comrie B. (2005). “Endangered numeral systems,” in *Bedrohte Vielfalt: Aspekte des Sprach(en)tods* [Endangered Diversity: Aspects of Language Death] eds Wohlgemuth J. Dirksmeyer T. (Berlin: Weißensee Verlag).

[B11] Deloche G. Seron X. (1982). From one to 1: an analysis of a transcoding process by means of neuropsychological data. *Cognition* 12 119–149. 10.1016/0010-0277(82)90009-9 6890429

[B12] Deloche G. Seron X. (1987). “Numerical transcoding: a general production model,” in *Mathematical Disabilities: A Cognitive Neuropsychological Perspecitve,* eds Deloche G. Seron X. (Hillsdale, NJ: Erlbaum), 37–179.

[B13] Dowker A. Bala S. Lloyd D. (2008). Linguistic influences on mathematical development: how important is the transparency of the counting system. *Philos. Psychol.* 21 523–538. 10.1080/09515080802285511

[B14] Dowker A. Lloyd D. (2005). “Linguistic influences on numeracy,” in *Mathematics in the Primary School*, eds Dowker A. Lloyd D. (Bangor: School of Education), 20–35.

[B15] Fuson K. Kwon Y. (1992). Korean children’s understanding of multidigit addition and subtraction. *Child Dev.* 63 491–506. 10.2307/1131494 1611949

[B16] Geary D. C. Fan L. Bow-Thomas C. C. (1992). Loci of ability differences comparing children from China and the United States. *Psychol. Sci.* 3 180–185. 10.1111/j.1467-9280.1992.tb00023.x

[B17] Göbel S. Moeller K. Pixner S. Kaufmann L. Nuerk H.-C. (2014). Language affects symbolic arithmetic in children: the case of number word inversion. *J. Exp. Child Psychol.* 119 17–25. 10.1016/j.jecp.2013.10.001 24269580

[B18] Helmreich I. Zuber J. Pixner S. Kaufmann L. Nuerk H.-C. Moeller K. (2011). Language effects on children’s nonverbal number line estimations. *J. Cross Cult. Psychol.* 42 598–613. 10.1177/0022022111406026

[B19] Hess R. D. Azuma H. (1991). Cultural support for schooling: contrasts between Japan and the United States. *Educ. Res.* 20 2–8. 10.3102/0013189X020009002

[B20] Holloway I. D. Ansari D. (2009). Mapping numerical magnitudes onto symbols: the numerical distance effect and individual differences in children’s math achievement. *J. Exp. Child Psychol.* 103 17–29. 10.1016/j.jecp.2008.04.001 18513738

[B21] Imbo I. Vanden Bulcke C. De Brauwer J. Fias W. (2014). Sixty-four or four-and-sixty? The influence of language and working memory on children’s number transcoding. *Front. Psychol.* 5:313 10.3389/fpsyg.2014.00313 PMC3990049 24782811

[B22] Kaufmann L. Handl P. Thoeny B. (2003). Evaluation of a numeracy intervention program focusing on basic numerical knowledge and conceptual knowledge: a pilot study. *J. Learn. Disabil.* 36 564–573. 10.1177/00222194030360060701 15493438

[B23] Krinzinger H. Gregoire J. Desoete A. Kaufmann L. Nuerk H. C. Willmes K. (2011). Differential language effects on numerical skills in second grade. *J. Cross Cult. Psychol.* 42 614–629. 10.1177/0022022111406252

[B24] Levine S. Jordan N. C. Huttenlocher J. (1992). Development of calculation abilities in young children. *J. Exp. Child Psychol.* 53 72–103. 10.1016/S0022-0965(05)80005-0 1545190

[B25] Link T. Schwarz E. J. Huber S. Fischer U. Nuerk H. C. Cress U. (2014). Mathe mit der Matte - Verkörperlichtes Training basisnumerischer Kompetenzen. *Z. Erziehungswiss.* 17 257–277. 10.1007/s11618-014-0533-2

[B27] Miller K. F. Kelly M. Zhou X. (2005). “Learning mathematics in China and the United States,” in *Handbook of Mathematical Cognition*, ed. Campbell J. I. D. (New York: Psychology press), 163–178.

[B28] Miller K. F. Smith C. M. Zhu J. Zhang H. (1995). Preschool origins of cross-national differences in mathematical competence: the role of number-naming systems. *Psychol. Sci.* 6 56–60. 10.1111/j.1467-9280.1995.tb00305.x

[B29] Miura I. T. Kim C. C. Chang C.-M. Okamoto Y. (1988). Effects of language characteristics on children´s cognitive representation of number: cross-national comparisons. *Child Dev.* 59 1445–1450. 10.2307/1130659

[B30] Miura I. T. Okamoto Y. (1989). Comparisons of U.S. and Japanese first graders’ cognitive representation of number and understanding of place value. *J. Educ. Psychol.* 81 109–114. 10.1037/0022-0663.81.1.109

[B31] Miura I. T. Okamoto Y. (2003). “Language supports for mathematics understanding and performance,” in *The Development of Arithmetic Concepts and Skills,* eds Baroody A. Dowker A. (Mahwah, NJ: Lawrence Erlbaum Associates), 229–242.

[B32] Miura I. T. Okamoto Y. Kim C. C. Chang C.-M. Steere M. Fayol M. (1994). Comparisons of children’s cognitive representation of number: China, France, Japan, Korea, Sweden, and the United States. *Int. J. Behav. Dev.* 17 401–411. 10.1177/016502549401700301

[B33] Miura I. T. Okamoto Y. Vlahovic-Stetic V. Kim C. C. Han J. H. (1999). Language supports for children´ s understanding of numerical fractions: cross-national comparisons. *J. Exp. Child Psychol.* 74 356–365. 10.1006/jecp.1999.2519 10552923

[B34] Moeller K. Pixner S. Kaufmann L. Nuerk H.-C. (2009). Children’s early mental number line: logarithmic or rather decomposed linear? *J. Exp. Child Psychol.* 103 503–515. 10.1016/j.jecp.2009.02.006 19328495

[B35] Moeller K. Pixner S. Zuber J. Kaufmann L. Nuerk H.-C. (2011). Early place-value understanding as a precursor for later arithmetic performance – a longitudinal study on numerical development. *Res. Dev. Disabil.* 32 1837–1851. 10.1016/j.ridd.2011.03.012 21498043

[B36] Moeller K. Shaki S. Goebel S. M. Nuerk H.-C. (2015) Language influences number processing – a quadrilingual study. *Cognition* 136 150–155 10.1016/j.cognition.2014.11.003 25497523

[B37] Moura R. Wood G. Pinheiro-Chagas P. Lonnemann J. Krinzinger H. Willmes K. (2013). Transcoding abilities in typical and atypical mathematics achievers. *J. Exp. Child Psychol.* 116 707–727. 10.1016/j.jecp.2013.07.008 24007971

[B38] Ng S. S. N. Rao N. (2010). Chinese number words, culture, and mathematics learning. *Rev. Educ. Res.* 80 180–206. 10.3102/0034654310364764

[B39] Perry M. VanderStroep S. W. Yu S. L. (1993). Asking questions in first-grade mathematics classes: potential influences on mathematical thought. *J. Educ. Psychol.* 85 31–40. 10.1037/0022-0663.85.1.31

[B40] Power R. J. D. Dal Martello M. F. (1990). The dictation of Italian numerals. *Lang. Cognitive Proc.* 5 237–254. 10.1080/01690969008402106

[B41] Power R. Dal Martello M. F. (1997). From 834 to eighty thirty four: the reading of arabic numerals by seven-year-old children. *Math. Cognit.* 3 63–85. 10.1080/135467997387489

[B42] Pixner S. Zuber J. Hermanova V. Kaufmann L. Nuerk H.-C. Moeller K. (2011). One language, two number-word systems and many problems: numerical cognition in the Czech language. *Res. Dev. Disabil.* 32 2683–2689. 10.1016/j.ridd.2011.06.004 21763104

[B43] Salillas E. Carreiras M. (2014). Core number representations are shaped by language. *Cortex* 52 1–11. 10.1016/j.cortex.2013.12.009 24607264

[B45] Seron X. Fayol M. (1994). Number transcoding in children: a functional analysis. *Brit. J. Dev. Psychol.* 12 281–300. 10.1111/j.2044-835X.1994.tb00635.x

[B46] Song M.-J. Ginsburg H. P. (1987). The development of informal and formal mathematics thinking in Korean and U.S. children. *Child Dev.* 58 1286–1296.

[B47] Stevenson H. W. Lee S. Y. (1990). Contexts of achievement: a study of American, Chinese, and Japanese children. *Monogr. Soc. Res. Child Dev.* 55 1–123. 10.2307/1166090 2342493

[B48] Stevenson H. W. Lee S. Y. Stigler J. W. (1987). Mathematics achievement of Chinese, Japanese, and American Children. *Science* 231 693–699. 10.1126/science.3945803 3945803

[B49] Stevenson H. W. Nerison-Low R. (2000). *To Sum it Up: Case Studies of Education in Germany, Japan, and the United States*. Philadelphia, PA: Mid-Atlantic Eisenhower Consortium for Mathematics and Science Education, National Institute on Student Achievement, Curriculum, and Assessment.

[B50] Stevenson H. W. Stigler J. W. Lee S. Y. Lucker G. W. Kitamura S. Hsu C. C. (1985). Cognitive performance and academic achievement of Japanese, Chinese, and American children. *Child Dev.* 56 718–734. 10.2307/1129761 4006575

[B51] Stigler J. W. Lee S. Y. Stevenson H. W. (1987). Mathematics classrooms in Japan, Taiwan, and the United States. *Child Dev.* 58 1272–1285. 10.2307/1130620 3665645

[B52] Towse J. N. Saxton M. (1997). Linguistic influences on children’s number concepts: methodological and theoretical considerations. *J. Exp. Child Psychol.* 66 362–375. 10.1006/jecp.1997.2389 9299080

[B54] Vasilyeva M. Laski E. V. Ermakova A. Lai W. F. Jeong Y. Hachigian A. (2015). Reexamining the language account of cross-national differences in base-10 number representations. *J. Exp. Child Psychol.* 129 12–25. 10.1016/j.jecp.2014.08.004 25240152

[B55] Zuber J. Pixner S. Moeller K. Nuerk H.-C. (2009). On the language-specificity of basic number processing: transcoding in a language with inversion and its relation to working memory capacity. *J. Exp. Child Psychol.* 102 60–77. 10.1016/j.jecp.2008.04.003 18499120

